# The Impact of Socioeconomic Factors on Knowledge, Attitudes, and Practices of Dog Owners on Dog Rabies Control in Thailand

**DOI:** 10.3389/fvets.2021.699352

**Published:** 2021-08-19

**Authors:** Sith Premashthira, Sarin Suwanpakdee, Weerapong Thanapongtharm, Onpawee Sagarasaeranee, Weerakorn Thichumpa, Chayanin Sararat, Anuwat Wiratsudakul

**Affiliations:** ^1^Department of Livestock Development, Bangkok, Thailand; ^2^Department of Clinical Sciences and Public Health, and the Monitoring and Surveillance Center for Zoonotic Diseases in Wildlife and Exotic Animals, Faculty of Veterinary Science, Mahidol University, Nakhon Pathom, Thailand; ^3^Department of Tropical Hygiene, Faculty of Tropical Medicine, Mahidol University, Bangkok, Thailand; ^4^Biophysics Group, Department of Physics, Faculty of Science, Mahidol University, Bangkok, Thailand

**Keywords:** epidemiology, KAP, public education, willingness to pay, zoonosis

## Abstract

Rabies is a deadly zoonotic disease responsible for almost 60,000 deaths each year, especially in Africa and Asia including Thailand. Dogs are the major reservoirs for rabies virus in these settings. This study thus used the concept of knowledge, attitudes, and practices (KAP) to identify socioeconomic factors that contribute to the differences in the canine rabies occurrences in high and low-risk areas which were classified by a Generalized Additive Model (GAM). Multistage sampling was then applied to designate the study locations and a KAP-based questionnaire was used to retrieve data and relevant perspectives from the respondents. Based on the responses from 476 participants living across four regions of Thailand, we found that the knowledge of the participants was positively correlated with their behaviors but negatively associated with the attitudes. Participants who are male, younger, educated at the level of middle to high school, or raising more dogs are likely to have negative attitudes but good knowledge on rabies prevention and control whereas farmers with lower income had better attitudes regardless of their knowledge. We found that people in a lower socioeconomic status with a lack of knowledge are not willing to pay at a higher vaccine price. Public education is a key to change dog owners' behaviors. Related authorities should constantly educate people on how to prevent and control rabies in their communities. Our findings should be applicable to other countries with similar socioeconomic statuses.

## Introduction

Rabies is a deadly zoonotic disease caused by Lyssaviruses belonging to the family Rhabdoviridae of the order Mononegavirales (1). With an almost 100% mortality rate, infected individuals are always fatal once symptoms develop (2). A wide variety of mammals were reported to harbor the virus, for example, bats, dogs, raccoons, and skunks (3). However, most of the human rabies cases are dog-mediated. It was estimated that canine rabies is responsible for around 59,000 human deaths annually and most of the endemic countries are located in Asia and Africa (4). Among those countries, Thailand had recently suffered from an unprecedented outbreak of rabies in animals. From the national active and passive surveillance program in which majority of the samples were passively collected, the Department of Livestock Development, Thailand (DLD) found positive results to rabies examination (fluorescent antibody technique and mouse inoculation test) at 15.3% (1,476/9,643) and 5.1% (377/7,321) in 2018 and 2019, respectively (5). Although the trend of rabies outbreaks is decreasing, there are still ongoing outbreaks in both humans and animals. In 2020, three human rabies deaths had been notified in three different provinces whereas the animal rabies cases had been recorded in 36 out of 77 provinces of Thailand. Dogs are the most active animals in the spread of the virus in these communities (5). Among the samples tested by DLD in 2020, most were retrieved from dogs (62.8%; 4,428/7,056). The submitted samples were primarily collected from animals suspected of symptoms of rabies. However, some died from other causes, such as car crashes and their samples were then sent to the laboratory as a part of the active surveillance.

In Thailand, dog owners must have a full responsibility for their dogs. Legally, the dogs must be vaccinated against rabies and kept from biting others. However, we do have a situation where stray dogs are fed without anyone claiming ownership. To get an overall herd immunity for rabies, DLD conducts a mass vaccination campaign annually for both owned and stray dogs.

The socioeconomic status of people living in a certain setting can directly affect three important aspects of health namely health care, environmental exposure, and health behavior (6). The problem of socioeconomic health disparities has been previously observed in many countries across the globe, for instance, United States (7), South Africa (8), Japan (9), and Indonesia (10). In Thailand, socioeconomic disparities among people classified in different social classes were previously pointed out. Several related health problems have been raised such as hypertension (11) and chronic respiratory diseases (12). Such disparity may also affect how people perceive and behave during rabies outbreaks in Thai communities. Regarding the rabies problem, it was found in a previous study that socioeconomic factors were associated with human rabies infection in China (13). It is worth exploring the influences of socioeconomics on rabies situations in Thailand.

The study of knowledge, attitudes, and practices (KAP) is on a curious basis that whether the increase of knowledge is correlated with attitudes and practices. The observed relationships will be then applied to tailor relevant policies to mitigate health problems (14). KAP has been previously used in the study of different infectious diseases, for instance, Leptospirosis (15), Brucellosis (16), and rabies (17–19). KAP would be an effective tool in exploring more insights into the behavioral aspects of dog owners. More understanding of these anthropogenic factors is helpful to target the right interventions to the right groups of people. In Indonesia, it was found that the attitude of dog owners was significantly associated with the intention to participate in a rabies control measure (20). Therefore, more understanding on the attitudes would be helpful in policy recommendation.

The present study, therefore, exploited KAP to identify the factors, especially on the socioeconomic aspects, relevant to the dog owners that contribute to the differences in the dog rabies occurrences in high and low-risk areas located in four different regions of Thailand.

## Materials and Methods

### Selection of Study Sites

A spatial risk map of dog rabies infection was produced at the district level of Thailand. Briefly, we used a Generalized Additive Model (GAM) to quantify the relationship between rabies occurrences and a set of explainable factors at the sub-district level including human population, dog population, cattle population, length of the road, and distance from the case locations to the country border. Then, the model was used to predict the probability of rabies occurrences in all sub-districts in the country. Finally, the rabies risk map at the district level was produced by averaging the predicted values of the sub-district level and classified into three risk levels namely low, medium and high. All modeling processes were performed with the package “mgcv” in program R version 4.0.3 (R Core Team, Vienna, Austria).

Multistage sampling was then used to designate the study locations. To deal with the culture and socioeconomic diversity, we performed our field investigation in four regions of Thailand. In each region, the province with the highest risk was chosen. Within each province, one high-risk and another low-risk districts were purposely selected.

### KAP Survey

A KAP-based questionnaire was prepared and validated before use by the experts for the consistency of the content in the questions. The questions were divided into four parts including (1) demographic data of the respondents (14 questions), (2) knowledge (11 questions), (3) attitudes (11 questions), and practices (11 questions). The questionnaires were distributed equally to each district. The original questionnaire was prepared in Thai. We translated it into English and attached in the Supplementary Material. The overview of the questions we asked was summarized in Table 1.

**Table 1:** Overview of the KAP questions.

**Category**	**Questions**
Demographic data of the respondents	(1) district where the residence is located (2) age (3) gender (4) education (5) main occupation (6) religion (7) income (8) type and number of pets (9) house ownership status (10) fence and gate (11) number of house members and their age (12) community roles
Knowledge	The questions asked about knowledge on (1) rabid animal species (2) seasonal restriction of the rabies outbreak (3) clinical signs (4) transmission (5) treatment of infected animals or humans (6) prevention by vaccine (7) first shot of dog vaccination (8) repeated vaccination annually (9) vaccine retention (10) vaccination in sick animals (11) prevention by ears and tail cutting
Attitudes	The questions asked about opinion on (1) harmfulness of rabies to pets and humans (2) annual vaccination (3) destruction of bitten dogs (4) destruction of stray dogs (5) sterilization (6) responsibility for the cost of vaccination (7) notification of suspected rabid dogs (8) identification and registration of dogs and cats (8) offense to the law in case of dog releasing in public place (9) stray dog quarantine
Practices	(1) rabies vaccination history for their dogs (2) vaccination practices (3) vaccine price willing to pay (4) sterilization of dogs (5) area restriction of dogs (6) dog saliva exposure avoiding (7) experience of encountering rabid dogs and notification to government agency (8) history of rabid dog exposure of their dogs and their action taking (9) rabies case-finding action (10) channels to receive rabies outbreak information

The target population was the dog owners who had at least one dog at home. The sample size required for the survey was calculated following the formula proposed by Cochran, 1977 (21) with 95% confidence interval and 5% margin of error. We set the sample proportion at 16.8% as suggested by a previous study on KAP in Thailand (22). The participants were face-to-face interviewed and the responses were filled into ODK-open software (https://opendatakit.org/) and stored on the cloud database. The data was, later on, downloaded for further analysis.

### Data Analysis

The demographic data were explored by descriptive analysis and K-means clustering. Cluster analysis helps identify structures within the data including their KAP scores. The homogenous groups of socioeconomic clusters of dog owners were identified. The principle of minimizing intra-cluster distance and maximizing the inter-cluster distance facilitated us to distinguish behavior in each study group (23). In our analysis, we use non-hierarchical cluster analysis or K-Means clustering. The logistic regression was applied to explore the association between factors and risk of areas (low and high-risk areas). Also, the multiple logistic regression was analyzed at the end to fit the best model. The scores of attitudes, knowledge, and practices were compared between both areas. Our scoring criteria were detailed in Supplementary Table 1. The correlations of knowledge, attitude and practice scores were analyzed using Spearman's correlation coefficient. The cut-off for the statistical difference was set at a *p*-value < 0.05. All data were analyzed with the packages “lmtest” and “zoo” in program R version 4.0.3 (R core team, Vienna, Austria) and the K-means commands equipped in the SPSS software version 19 (IBM Corp. Released 2010. IBM SPSS Statistics for Windows, Version 19.0. Armonk, NY: IBM Corp.) was used to classify clusters. The Euclidean distances were then calculated to assess the distance between the final clusters.

## Results

### Study Locations

**Figure 1:**
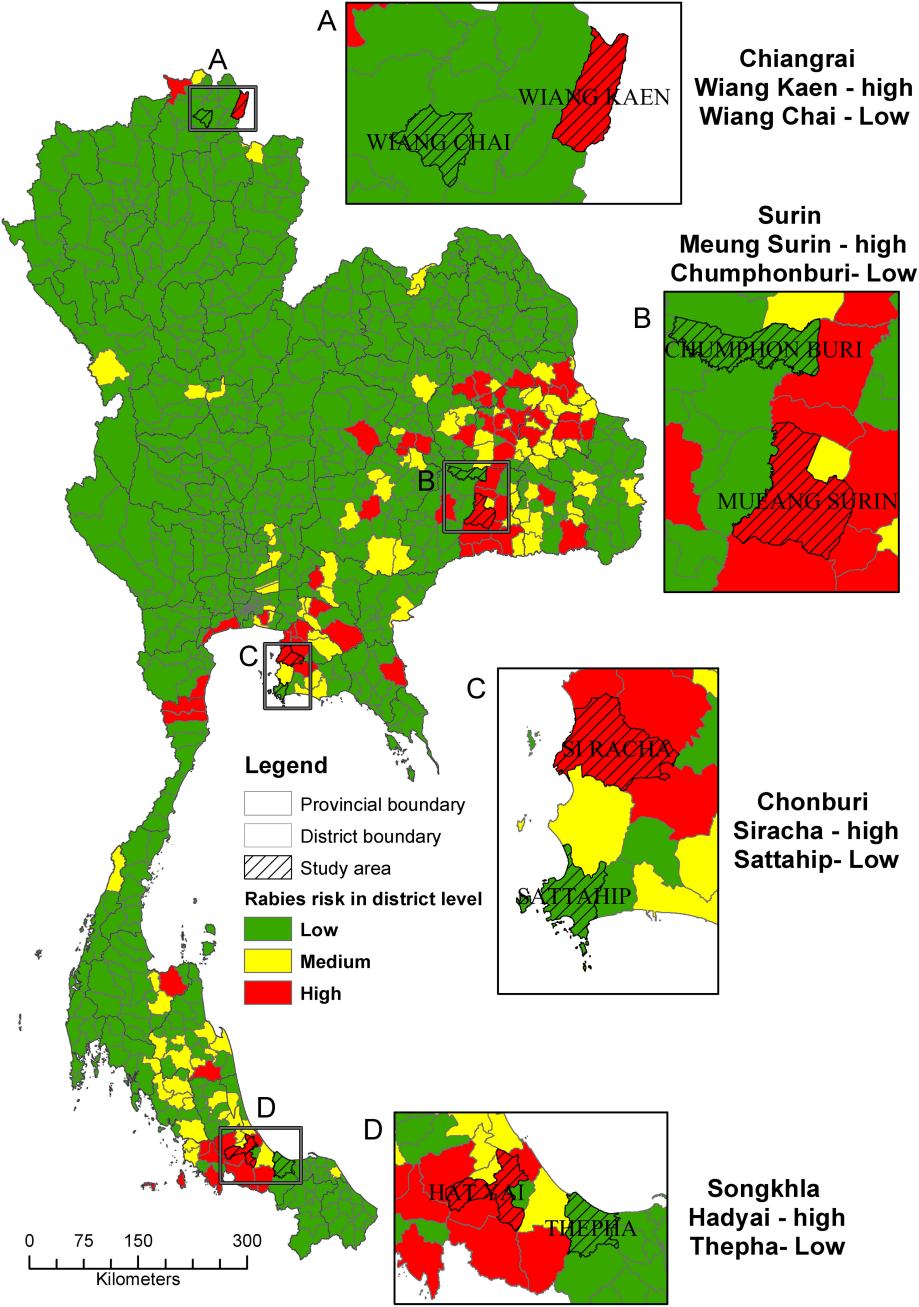
A map depicting the risk level of canine rabies transmission at district level for the selection of study areas (shaded; **A–D**) in the four main regions of Thailand.

We classified 926 districts in Thailand according to the risk of rabies outbreak occurrence as 65 high-risk, 101 medium-risk, and 760 low-risk districts, respectively. Within the four main regions, the provinces that contained the highest number of high-risk districts in each region were Chiang Rai in the North (two districts), Surin in the Northeast (nine districts), Chon Buri in the Central (five districts), and Songkhla in the South (six districts). One high-risk and one low-risk districts located in these provinces were then chosen as our study sites (Figure 1).

### Demographic Data of the Respondents

In total, we had 476 participants involved in our questionnaire survey, of which 243 (51.1%) resided in the high-risk areas. The average age of the participants is 53 years (range: 18–93). Overall, the majority of the participants are Buddhist females and educated to the level of primary school. The main occupation is farmer with an average income per month of <10,000 Thai Baht (≈ 320.5 USD). The average number of family members in their households is 4.3 persons. These participants keep around 2.6 dogs at home with an average raising experience of 7.1 years. Two-third of their dogs are mixed breed and raised without fencing. These demographic data are summarized in Table 2.

**Table 2:** Demographic data of the KAP respondents.

**Variables**	**Number (Percentage, 95% CI)**		
	**High-risk areas**	**Low-risk areas**	**Total**
Number of respondents	243	233	476
**Age (year)**			
- Up to 20	8 (3.3%, 1.4–6.4%)	0 (0.0%, 0.0–0.0%)	8 (1.7%, 0.7–3.3%)
−21–40	46 (18.9%, 14.2–24.4%)	47 (20.2%, 15.2–25.9%)	93 (19.5%, 16.1–23.4%)
−41–60	116 (47.7%, 41.3–54.2%)	100 (42.9, 36.5–49.5%)	216 (45.4%, 40.8–50.0%)
- Over 60	73 (30.0%, 24.4–36.2%)	86 (36.9%, 30.7–43.5%)	159 (33.4%, 29.2–37.8%)
**Gender**			
- Female	166 (68.3%, 62.1–74.1%)	162 (69.5%, 63.2–75.4%)	328 (68.9%, 64.5–73.0%)
- Male	77 (31.7%, 25.9–37.9%)	71 (30.5%, 24.6–36.8%)	148 (31.1%, 26.0–34.4%)
**Education**			
- Below primary	32 (13.2%, 9.2–18.1%)	8 (3.4%, 1.5–6.7%)	40 (8.4%, 6.1–11.3%)
- Primary	111 (45.7%, 39.3–52.2%)	120 (51.5%, 44.9–58.1%)	231 (48.5%, 44.0–53.1%)
- Middle/high	55 (22.6%, 17.5–28.4%)	56 (24.0%, 18.7–30.1%)	111 (23.3%, 19.6–27.4%)
- Vocational	12 (4.9%, 2.6–8.5%)	24 (10.3%, 6.7–14.9%)	36 (7.6%, 5.4–10.3%)
- Bachelor or higher	33 (13.6%, 9.5–18.5%)	25 (10.7%, 7.1–15.4%)	58 (12.2%, 9.4–15.5%)
**Main occupation**			
- Farmer	90 (37.0%, 30.1–43.4%)	108 (46.4%, 39.8–53.0%)	198 (41.6%, 37.1–46.2%)
- Housewife	48 (19.8%, 14.9–25.3%)	40 (17.2%, 12.6–22.6%)	88 (18.5%, 15.1–22.3%)
- Merchant	54 (22.2%, 17.2–28.0%)	27 (11.6%, 7.8–16.4%)	81 (17.0%, 13.8–20.7%)
- Freelance	22 (9.1%, 5.8–13.4%)	17 (7.3%, 4.3–11.4%)	39 (8.2%, 5.9–11.0%)
- Public servant	12 (4.9%, 2.6–8.5%)	22 (9.4%, 6.0–14.0%)	34 (7.1%, 5.0–9.8%)
- Others	17 (7.0%, 4.1–11.0%)	19 (8.1%, 5.0–12.4%)	36 (7.6%, 5.4–10.3%)
**Religion**			
- Buddhist	214 (88.1%, 83.3–92.0%)	227 (97.4%, 94.5–99.1%)	441 (92.6%, 89.9–94.8%)
- Christ	12 (4.9%, 2.6–8.5%)	5 (2.1%, 0.7–4.9%)	17 (3.6%, 2.1–5.7%)
- Islam	3 (1.2%, 0.3–3.6%)	1 (0.4%, 0.0–2.4%)	4 (0.8%, 0.2–2.1%)
- Traditional ghost beliefs	13 (5.3%, 2.9–9.0%)	0 (0.0%, 0.0–0.0%)	13 (2.7%, 1.5–4.6%)
- No religion	1 (0.4%, 0.0–2.3%)	0 (0.0%, 0.0–0.0%)	1 (0.2%, 0.0–1.2%)
**Income per month (THB)**			
- Up to 10,000	115 (47.3%, 40.9–53.8%)	129 (55.4%, 48.7–61.9%)	244 (51.3%, 46.7–55.8%)
−10,001–20,000	68 (28.0%, 22.4–34.1%)	51 (21.9%, 16.8–27.8%)	119 (25.0%, 21.2–29.1%)
−20,001–30,000			
- Over 30,000	18 (7.4%, 4.5–11.5%)	27 (11.6%, 7.8–16.4%)	45 (9.5%, 7.0–12.5%)
(USD 1 ≈ THB 31.2)	42 (17.3%, 12.8–22.6%)	26 (11.2%, 7.4–15.9%)	68 (14.3%, 11.3–17.8%)
**Animal keeping**			
- Number of dogs	Mean = 2.8 (SD = 4.9)	Mean = 2.4 (SD = 2.1)	Mean = 2.6 (SD = 3.8)
- Breed
Mixed	145 (59.7%, 53.2–65.9%)	156 (67.0%, 60.5–73.0%)	301 (63.2%, 58.7–67.6%)
Poodle	25 10.3%, 6.8–14.8%)	12 (5.2%, 2.7–8.8%)	37 (7.4%, 5.5–10.6%)
Bangkaew	13 (5.4%, 2.9–9.0%)	10 (4.3%, 2.1–7.8%)	23 (4.8%, 3.1–7.2%)
- Years of experience	Mean = 7.3 (SD = 7.8)	Mean = 7.0 (SD = 6.9)	Mean = 7.1 (SD = 7.4)
**Type of house ownership**			
- Owned	217 (89.3%, 84.7–92.9%)	224 (96.1%, 92.8–98.2%)	441 (92.6%, 89.9–94.8%)
- Rent	11 (4.5%, 2.3–8.0%)	3 (1.3%, 0.3–3.7%)	14 (2.9%, 1.6–4.9%)
- Dormitory	1 (0.4%, 0.0–2.3%)	1 (0.4%, 0.0–2.4%)	2 (0.4%, 0.1–1.5%)
- Others	14 (5.8%, 3.2–9.5%)	5 (2.2%, 95%CI 0.7–4.9%)	19 (4.1%, 95%CI 2.4–6.2%)
**House fencing**			
- Yes	87 (35.8%, 95%CI 29.8–42.2%)	81 (34.8%, 28.7–41.3%)	168 (35.3%, 31.0–39.8%)
- No	156 (64.2%, 57.8–70.2%)	152 (65.2%, 58.7–71.3%)	308 (64.7%, 60.2–69.0%)
- Number of family member	Mean= 4.7 (SD = 2.7)	Mean = 4.0 (SD = 1.7)	Mean = 4.3 (SD = 2.3)

### KAP Results

**Figure 2:**
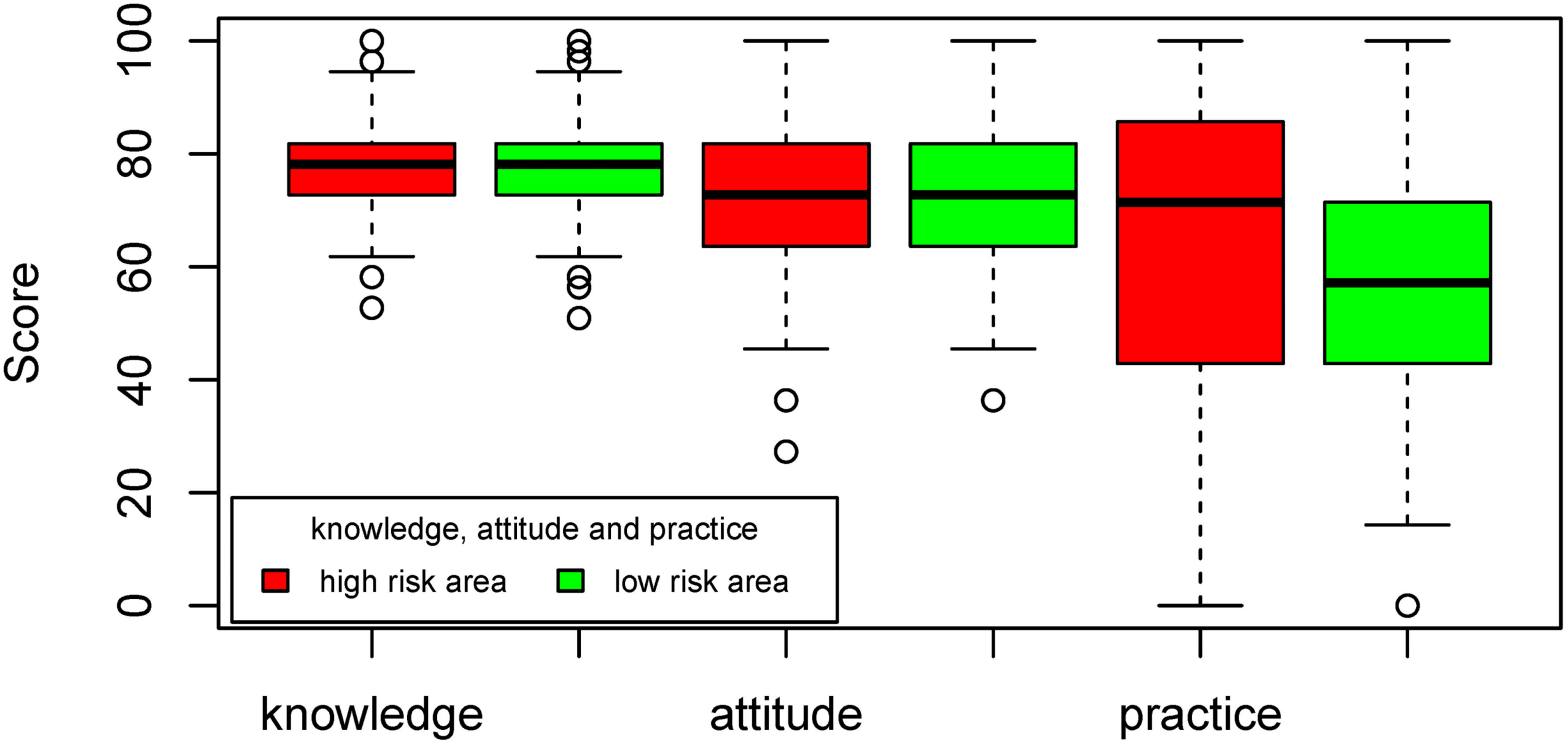
Comparison of the adjusted scores of attitudes, knowledge, and practices between the high and low-risk areas for rabies transmission.

We found no statistically significant differences in the knowledge, attitudes, and practices scores between high and low-risk areas (Figure 2). The descriptive statistics of the overall scores were depicted in the Supplementary Table 2. The KAP overview scores; however, before proving statistical significance, reflected that respondents living in the low-risk areas had better knowledge and attitude scores compared to those in the high-risk areas. However, this was not the case for the practice scores. The knowledge scores in the high-risk areas (averaged 8.12 ± 1.74) were lower than in the low-risk areas (averaged 8.28 ± 1.54). The attitude scores in the high-risk areas (averaged 42.36 ± 3.99) were slightly lower than the low-risk areas (averaged 42.53 ± 4.06). In contrast, the practice scores in the high-risk areas (averaged 4.54 ± 1.51) were higher than the low-risk areas (averaged 4.36 ± 1.48). The differences in the practices of the dog owners comparing between high and low-risk areas were identified in the questions related to vaccination practices and house fencing (Table 3). After controlling confounding factors, we found the differences only in the vaccination practices. Participants living in the high-risk areas were not likely to buy vaccines for their dogs but they preferred to get a free vaccination service provided by the government staff (odds ratio: 0.410; 95% CI: 0.22–0.76) (Table 4). In Table 5, we found an overall positive correlation between knowledge and practices and a negative correlation between knowledge and attitudes.

**Table 3:** Determinants of the level of risk areas for rabies (high/low) in the univariate analysis regarding the practices of the dog owners (*n* = 476).

**Practices of the dog owners**	**Odds ratio (95% CI)**	***P*-value**
**Frequency of dog rabies vaccination**		
- Annually	1.28 (0.65–2.54)	0.478
- Irregularly	1.04 (0.44–2.46)	0.927
- No vaccination	Reference	
**Vaccination practice**		
- Purchased vaccines by dog owner	0.40 (0.22–0.74)	0.004
- Paid service at veterinary hospitals/clinics	2.07 (1.04–4.35)	0.044
- Free service of government staff	Reference	
**First rabies vaccine shot at few-month-old dogs**		
- Yes	0.97 (0.68–1.40)	0.883
- No	Reference	
**Dog population control**		
- Yes	1.16 (0.80–1.68)	0.441
- No	Reference	
**House fencing**		
- Yes	1.58 (1.09–2.30)	0.017
- No	Reference	
**Avoiding dog saliva exposure**		
- Yes	1.06 (0.73–1.52)	0.768
- No	Reference	
**Experience of encountering rabid dogs**		
- Yes	1.16 (0.79–1.69)	0.457
- No	Reference	
**Notification of rabid dogs to government agency**		
- Yes	1.14 (0.57–2.28)	0.709
- No	Reference	
**Rabid dog exposure to their dogs**		
- Yes	1.39 (0.52–3.88)	0.515
- No	Reference	

**Table 4:** Determinants of the level of risk areas for rabies (high/low) in the logistic multivariable regression model regarding the practices of the dog owners (*n* = 476).

**Practices of the dog owners**	**OR (95% CI)**	***P*-value**
**Vaccination practice**		
Purchased vaccines by dog owner	0.41 (0.22–0.76)	0.005
Paid service at animal hospitals/clinics	1.79 (0.88–3.82)	0.12
Free service of government staff	Reference	
**House fencing**		
Yes	1.43 (0.95–2.14)	0.08
No	Reference	

**Table 5:** Spearman's correlation coefficient between knowledge, attitudes and practices of dog owners regarding rabies control in Thailand.

	**Knowledge**	**Attitudes**	**Practices**
Knowledge	1.000	−0.100^*^(0.030)	0.206^**^(0.000)
Attitudes	−0.100^*^(0.030)	1.000	0.037 (0.422)
Practices	0.206^**^ (0.000)	0.037 (0.422)	1.000

** *Correlation is significant at the 0.01 level (2-tailed)*.

* *Correlation is significant at the 0.05 level (2-tailed)*.

### Socioeconomic Clusters Regarding the KAP Scores

The sample population was clustered according to their KAP scores into three groups (Table 6) to compare socioeconomic factors affecting how dog owners control rabies in Thailand. The three classified clusters were: cluster 1—positive attitudes but poor knowledge, cluster 2—negative attitudes but good knowledge, and cluster 3—positive attitudes and good knowledge. The statistically significant difference between clusters was found in the knowledge and attitudes, but not in the practice scores. We found no difference between the clusters in the main indicator of this study, that is, living in the high and low-risk areas. Nevertheless, the statistical difference was denoted among clusters for the factors of age, gender, education, occupation, income, number of dogs raised, the average age of the youngest family member, and the average age of the oldest family member.

**Table 6:** KAP score clusters to describe socioeconomic status of dog owners regarding rabies control in Thailand.

**Factors**	**Cluster percent count percent**			
	**1**	**2**	**3**	**Significance^a^**
Number of cases in the cluster	99	105	272	
Group describe	Positive attitudes but poor knowledge	Negative attitudes but good knowledge	Positive attitudes and good knowledge	
Total practice score	4.46	4.42	4.46	0.964^ns^
Total attitude score	47.90	37.11	42.51	0.000^**^
Total knowledge score	7.67	8.35	8.33	0.001^**^
Risk area	High 50.5%	High 54.3%	High 50.0%	0.751^ns^
	Low 49.5%	Low 45.7%	Low 50.0%	
Region	South 28.3%	South 32.4%	NE 29.0%	0.055^ns^
	NE 26.3%	East 30.5%	South 26.5%	
	North 26.3%	North 23.8%	North 23.2%	
	East 19.2%	NE 13.3%	East 21.3%	
Average age (year)	58.05	50.21	52.16	0.000^**^
Gender	Female 73.7%	Female 55.2%	Female 72.4%	0.003^**^
	Male 26.3%	Male 44.8%	Male 27.6%	
Education	Primary 62.6%	Primary 41.0%	Primary 46.3%	0.038^*^
	Middle to high 17.2%	Middle to high 33.3%	Middle to high 21.7%	
	Bachelor to higher 8.1%	Bachelor to higher 12.4%	Bachelor to higher 13.6%	
Occupation	Farmer 45.5%	Farmer 32.4%	Farmer 43.8%	0.008^**^
	Housewife 22.2%	Housewife 21.0%	Housewife 16.2%	
	Merchant 16.2%	Merchant 16.2%	Merchant 17.6%	
Income	Below 10,000 = 50.5%	Below 10,000 = 38.1%	Below 10,000 = 56.6%	0.006^**^
	10,000–20,000 = 30.3%	10,000–20,000 = 26.7%	10,000–20,000 = 22.4%	
	20,000–30,000 = 10.1%	20,000–30,000 = 16.2%	20,000–30,000 = 6.6%	
	Over 30,000 = 9.1%	Over 30,000 = 19.0%	Over 30,000 = 14.3%	
Number of dogs raised	2.32	3.73	2.2	0.002^**^
The average age of the youngest family member	26.42	21.65	19.10	0.009^**^
The average age of the oldest family member	62.78	57.94	61.66	0.039^*^
Rabies vaccine price that willing to pay	76.64	73.71	74.08	0.927^ns^

a *Statistically significant differences across groups were tested using Pearson's χ^2^ test (*

* *indicates statistically significant relationships for p < 0.05*,

** *for p < 0.01 and*

ns *stands for non-significant relationships)*.

### Socioeconomic Cluster Regarding Income, Education, and Willingness to Pay for Rabies Vaccines

After removing missing data, 470 dog owners were clustered regarding their income, education, and rabies vaccine prices that they are willing to pay. Three clusters were designated namely (1) willing to pay the highest cost of the vaccine, (2) moderate cost of the vaccine and (3) lowest cost of the vaccine (Table 7). The average prices that the participants in each cluster are willing to pay were 251.60, 91.09, and 38.58 Thai Baht, respectively. The differences between these three clusters were identified for the main indicator of this study (living in high and low-risk areas). Participants living in the high-risk areas are willing to pay more compared to those who lived in the low-risk areas. Besides the risk levels, other factors that significantly influence the willingness to pay are geographical region, age, education, occupation, and income.

**Table 7:** Clusters of income, education, and rabies vaccine price that willing to pay of dog owners regarding rabies control in Thailand.

**Factors**	**Cluster percent count percent**			
	**1**	**2**	**3**	**Significance^**a**^**
Number of cases in the cluster	31	196	243	
Group Describe	willing to pay the highest cost of the vaccine	willing to pay the moderate cost of the vaccine	willing to pay the cheapest cost of the vaccine	
Rabies vaccine price that willing to pay (Baht)	251.60	91.09	38.58	0.000^**^
Total practice score	4.32	4.46	4.34	0.100^ns^
Total attitude score	43.35	41.93	42.71	0.054^ns^
Total knowledge score	8.35	8.18	8.00	0.046^*^
Risk area	High 74.2%	High 53.1%	High 46.0%	0.013^*^
	Low 25.8%	Low 46.9%	Low 53.1%%	
Region	North 3.26%	North 17.9%	North 32.1%	0.000^**^
	NE 32.3%	NE 9.7%	NE 37.0%	
	East 22.6%	East 32.1%	East 16.0%	
	South 41.9%	South 40.3%	South 14.8%	
Average age	49.65	50.95	55.19	0.004^**^
Gender	Female 74.2%	Female 72.4%	Female 65.0%	0.197^ns^
	Male 25.8%	Male 27.6%	Male 35.0%	
Education	Primary 35.5%	Primary 42.3%	Below primary 10.7%	0.000^**^
	Middle to high 38.7%	Middle to high 24.0%	Primary 56.0%	
	Bachelor to higher 22.6%	Bachelor to higher 17.9%	Middle to high 21.0%	
Occupation	Farmer 32.3%	Farmer 33.2%	Farmer 50.2%	0.000^**^
	Housewife 22.6%	Merchant 22.4%	Housewife 18.1%	
	Merchant 22.6%	Housewife 18.9%	Merchant 11.1%	
Income	Below 10,000 = 25.8%	Below 10,000 = 43.46%	Below 10,000 = 61.3%	0.000^**^
	10,000–20,000 = 48.4%	10,000–20,000 = 25.0%	10,000–20,000 = 21.8%	
	20,000–30,000 = 9.7%	20,000–30,000 = 11.2%	20,000–30,000 = 8.2%	
	Over 30,000 = 16.1%	Over 30,000 = 20.4%	Over 30,000 = 8.6%	
Number of dogs raised	1.96	2.24	2.91	0.120^ns^

a *Statistically significant differences across groups were tested using Pearson's χ^2^ test (*

* *indicates statistically significant relationships for p < 0.05*,

** *for p < 0.01 and*

ns *stands for non-significant relationships)*.

## Discussion

The present study used KAP techniques to cross-sectionally explore socioeconomic impacts on the spread of rabies virus among dog populations in the high and low-risk areas in Thailand. Subsequently, different statistical methods were employed to analyze socioeconomic factors that may contribute to rabies transmission.

We found that the high-risk areas for rabies propagation were identified in all regions of Thailand. However, the majority of the risky areas were disproportionately found in the Northeastern region (Figure 1). Our finding was in line with a previous study (24) that identified the hotspots of the rabies outbreaks in the same region. What we found here and the findings of the previous study are only a descriptive aspect of the outbreaks. An in-depth analysis is needed to find out the factors contributing to this observation.

Interestingly, our findings pointed out a different practice on how people get their dogs vaccinated (Table 4). This reflects the difference in social responsibility among people living in high and low-risk areas. It may also explain why the number of rabies cases was higher in the high-risk areas as the dog owners, in case they were unable to bring the dogs to the clinics, usually wait for a free vaccination service provided by the government whereas people in the low-risk areas actively purchase the vaccines for their dogs. Geographically, the high-risk areas visited in this study are located more remotely compared to the low-risk areas. Some participants also complained that the governmental services had not reached their premises. This may result in a low level of herd immunity. Indeed, the local administrative organization (LOAs) in Thailand have been working closely with the DLD to facilitate the distribution of the rabies vaccines to reach the rural and remote communities. However, there are still some unreachable areas as we found in our study. The uses of local leaders and administrators have been evidenced in the prevention and control of infectious diseases, for example, in the cases of Corona Virus Disease (COVID-19) (25) and Ebola (26). This approach should also be applicable in the case of rabies.

In Table 5, we found that the knowledge of the participants was positively correlated with their behaviors but negatively associated with their attitudes. A similar finding was also denoted in a previous study on rabies in Congo that poor knowledge of general people can lead to malpractices in the community (27). Our findings may direct the rabies control policy to focus more on providing knowledge and information on rabies prevention and control to the public rather than trying to change their perceptions. Nonetheless, a significant difference in the educational level of participants living in high and low-risk areas was observed in this study. Compared to the low-risk areas, we found 3.03 times (95% CI: 1.23-8.09, *p*-value = 0.02) higher in the number of people educated below primary level compared to the number of people with the bachelor or higher education living in the high-risk areas. The lower educational level of people living in the high-risk areas also affects how people comprehend messages announced by the government. In a previous study carried out in India, it was found that the low level of formal education is inversely linked to the knowledge of farmers regarding zoonotic diseases (28). To improve fundamental education is helpful to increase knowledge on rabies control and change relevant practices accordingly.

According to Table 6, we found multiple socioeconomic factors significantly influencing the knowledge and attitudes of the dog owners toward rabies control in their settings whether they were living in high or low-risk areas. It seems that participants who were categorized as male, younger age, educated at the level of middle to high school or raising more dogs tend to have negative attitudes but good knowledge whereas farmers with lower income had a better attitudes compared to other occupations regardless of their knowledge. Our findings reflect the complexity of how socioeconomic status impacts what people know and how they think about the control of rabies in Thailand. As the majority of people living in the study areas are farmers with primary education, we recommend, again, related authorities to constantly provide knowledge on how to prevent and control rabies to the general public, especially those who own dogs. In the policy implementation, the areas with poor people, aged higher and educated at the lower level should be firstly prioritized.

Different socioeconomic status of people included in this study also impacts on how much they are willing to pay for a dose of rabies vaccine (Table 7). Overall, we found that people who are younger, with higher education or higher income tended to pay more for the rabies vaccines. Moreover, people with higher knowledge scores are more willing to pay higher prices (*p*-value = 0.046). In a previous study on the willingness to pay for social health insurance in Vietnam, it was found that people with more knowledge on the issue are willing to pay more (29). Besides, our findings indicate that people living in high-risk areas are willing to pay higher. It implies that people who face directly with a crisis are more aware of the danger and ready to pay higher for their safety. This circumstance was also observed in the case of COVID-19 that people having family members infected with the virus are more likely to pay for the vaccines (30). Nontheless, a contradict result was observed in our study. Participants living in the high-risk areas were usually wait for a free vaccination service whereas they are still willing to pay more. This might be related to the availability of the vaccines in the areas. This observation should be further investigated. The socioeconomic disparity has previously been pointed out regarding rabies problems (31, 32). For example, a study in Cameroon suggested that more wealthy people with better knowledge of rabies are more likely to seek medical treatment and post-exposure prophylaxis (33). The inequality of socioeconomic status of people living in different areas should be seriously considered in the tailoring of rabies prevention and control programs as well as designing public education campaigns and risk communication.

Like other studies, we faced some potential limitations. First, we tried to include people in all regions of Thailand. However, with limited resources, we carried out our survey in only eight districts across the country. There might still be some variations of the socioeconomic factors that were not identified. A future study extending to cover a larger geographical area is recommended. Moreover, the participants involved in this study were recruited purposively. An ideal random sample is not feasible as there is no official registration of the dog owners in Thailand. Nevertheless, the relevant authorities have been now working together to set the system up. With the animal registration system, a survey study like this would be performed more effectively. Besides, it would be also beneficial to the proper allocations of the resources related to rabies control such as vaccines. In this study, we identified an important factor that can directly contribute to the better control of rabies epidemics, that is, public education. The impact of public education on rabies prevention has been addressed in a previous study conducted in Azerbaijan. It was found that people participating in the rabies awareness campaign are more likely to get their pets vaccinated (34). Therefore, we should identify channels that are the most effective ways in conveying the knowledge and governmental message to the genal public. This will increase their awareness and help controling the problem in long run.

In conclusion, the canine rabies outbreak is a complex problem involving multiple socioeconomic factors. Public education is a key to change the owners' behaviors regarding the control of rabies in Thailand. Related authorities should rigorously and constantly educate people on how to prevent and control rabies in their settings. Our findings should also be applicable to other countries with similar socioeconomic status.

## Data Availability Statement

The raw data supporting the conclusions of this article will be made available by the authors, without undue reservation.

## Ethics Statement

The studies involving human participants were reviewed and approved by Mahidol University—Center of Ethical Reinforcement for Human Research (MU-CIRB 2019/157.0606). The patients/participants provided their written informed consent to participate in this study.

## Author Contributions

SP and AW conceived the study. SP, SS, OS, CS, and AW participated in the collection of field data. SS, WT, and SP conducted the statistical analyses. AW oversaw the study and coordinated the drafting of the article. AW, SS, and SP were the main reviewers. All authors contributed to the article and approved the submitted version.

## Conflict of Interest

The authors declare that the research was conducted in the absence of any commercial or financial relationships that could be construed as a potential conflict of interest.

## Publisher's Note

All claims expressed in this article are solely those of the authors and do not necessarily represent those of their affiliated organizations, or those of the publisher, the editors and the reviewers. Any product that may be evaluated in this article, or claim that may be made by its manufacturer, is not guaranteed or endorsed by the publisher.
