# Supplementary material for: The Impact of Socioeconomic Factors on Knowledge, Attitudes, and Practices of Dog Owners on Dog Rabies Control in Thailand
Source: Front Vet Sci. 2021 Aug 19;8:699352. doi: 10.3389/fvets.2021.699352 (PMC8416909; doi:10.3389/fvets.2021.699352)
Supplement: Supplementary file 1 [file Table_1.DOCX]

**Supplementary material 1**

**Questionnaire: Knowledge, attitude and practice on rabies control in different risk areas of rabies in dogs.**

Date/month/year interviewed…………………. Questionnaire number……….

**Part 1: General/demographic data**

**Statement – Please fill in the text with spaces or write a mark × into 🗖 in front of the texts which you think it matches you the most**

- 1. You live in………………. district ……….…….. province……….……..
  2. Your age is………. years old
  3. Gender 🗖male 🗖female
  4. Highest education background
- Lower than primary school 🗖 primary school 🗖 secondary school 🗖 vocational school 🗖 bachelor’s degree or higher

1.5 Main occupation 🗖 student 🗖 government official or state enterprise employee 🗖farmer 🗖own business 🗖housewife 🗖 private employee 🗖 freelance 🗖 others (please specify).....................

1.6 Your religion 🗖Buddhist 🗖 Christ 🗖 Islam 🗖 others (please specify).....................

1.7 Monthly income

🗖less than 10,000 baht 🗖10,000-20,000 baht 🗖20,001-30,000 baht🗖more than 30,000 baht

1.8 Animals raised in your house

🗖dogs: number ….......heads breed.......................................period of raised time..........................years

🗖cats: number ….......heads breed.......................................period of raised time..........................years

🗖other mammal please specify………… number ….......heads breed.......................................period of raised time..........................years

1.9 The manner in which you are raising the dog gated (Can prevent dogs from going out of the house) 🗖No 🗖Yes

1.10 What kind of house is your house? 🗖own 🗖rent 🗖dormitory/apartment 🗖others please specify...............

1.11 How many house member (including yourself? .....................persons

1.12 How old is your youngest house member?..................years

1.13 How old is your oldest house member?..................years

1.14 Do you have a role/ duty in community (can be official/unofficial roles such as volunteer) ?

🗖no 🗖yes, please specify your roles......................................

**Part 2 Practices in rabies prevention and control**

**Statement – Please write a mark × into 🗖 which you practice regularly that are most relevant to you and fill in the text with spaces**

**- The word “your dog/cat" has a meaning, including dog(s) – cat(s) raised in your residence**

2.1 Has your dog-cat been vaccinated against rabies?

🗖Never

🗖Yes, complete vaccination every year.

🗖Yes, but not complete every year.

If you answer yes, please specify type of vaccinator.

🗖 Veterinarian at Clinic or Animal Hospital

🗖 Mobile service vaccinator from Department of Livestock Development or other government agencies

🗖 You bought vaccine to vaccinate by yourself

🗖 Others, please specify...........................................

2.2 Did you bring your dog-cat to be vaccinated first time at the age 2-3 months?

🗖 Never 🗖 Yes

Service by

🗖 Veterinarian at Clinic or Animal Hospital

🗖 Mobile service vaccinator from Department of Livestock Development or other government agencies

🗖 You bought vaccine to vaccinate by yourself

🗖 Others, please specify...........................................

2.3 Usually, the rabies vaccination price offered at an animal hospital is about 80-100 baht / head. You think ………….. baht / head you are willing to pay for the rabies vaccine.

2.4 You control the number of your dog/cat, such as taking them to be sterilized.

🗖Never 🗖Ever Where do dog/cat get sterilized.?.........................

2.5 Do you limit the area of your dog/cat so as not to expose to stray dogs? 🗖No 🗖Yes

2.6 Do you avoid dog/cat saliva exposure? 🗖No 🗖Yes

2.7 Have you ever seen a dog/cat which is suspected of rabies? 🗖No 🗖Yes

2.8 If you ever found an animal suspected of rabies, did you notify the government agency? 🗖No 🗖Yes

2.9 Has your dog/cat been bitten by a dog/cat suspected of rabies?

🗖Never

🗖Yes, later I proceed 🗖vaccination 🗖restraining 🗖destruction 🗖other (please specify)............................

2.10 If you found your dog/cat or neighbored dog/cat that was sick to death, you proceed

🗖bury 🗖burn 🗖throw away the trash or the waste pile 🗖submit to laboratory at ......................................................

2.11 Who have you received information about the rabies outbreak? ( You could select more than one answers by put the rank number in the 🗖)

🗖administrative staff 🗖public health volunteer 🗖Department of Livestock Development staff 🗖village voice by line 🗖radio 🗖television 🗖internet 🗖billboard

🗖other (please specify)..................................

**Part 3 Attitudes in rabies control**

**Statement – Please write a mark × into** 🗖 **according to the idea that is most relevant to you.**

| **Attitude** | **Strongly agree** | **Agree** | **Unsure** | **Disagree** | **Strongly disagree** |
| --- | --- | --- | --- | --- | --- |
| 3.1 Rabies is a dangerous disease for pets and people. |  |  |  |  |  |
| 3.2 Rabies vaccination should be given to pets every year. |  |  |  |  |  |
| 3.3 Animals bitten by a rabid dog should be eliminated immediately. |  |  |  |  |  |
| 3.4 Rabies at risk animal such as stray dogs should not be eliminated. |  |  |  |  |  |
| 3.5 Dog/cat should be sterilized so as not to multiply and become burdensome. |  |  |  |  |  |
| 3.6 Government agencies should bear the cost of vaccinating pets, not their owners**.** |  |  |  |  |  |
| 3.7 You should not notify government officials If a sick animal that is not your own, is suspected of rabies. |  |  |  |  |  |
| 3.8 You should notify government officials If you find your own pet is suspected of rabies. |  |  |  |  |  |
| 3.9 Dog/cat should be registered and control the number of raising dog/cat. |  |  |  |  |  |
| 3.10 No offense under the law for people who bring dog/cat to release in public area. |  |  |  |  |  |
| 3.11 Government agencies should detain dog/cat strayed from their owners for detention. |  |  |  |  |  |

**Part 4 Knowledge in rabies control**

**Statement – Please write a mark × into only one 🗖 which you saw as correct.**

4.1 Rabies only occurs in dogs.

🗖 Right 🗖 Not right

4.2 Rabies only occurs in the summer.

🗖 Right 🗖 Not right

4.3 Some sick animals with lethargic illness can also be rabies.

🗖 Right 🗖 Not right

4.4 Rabies is mainly transmitted by animal bites or saliva exposure.

🗖 Right 🗖 Not right

4.5 Animals or people infected with rabies and show sickness can be cured.

🗖 Right 🗖 Not right

4.6 Vaccination of rabies in animals can prevent disease.

🗖 Right 🗖 Not right

4.7 Vaccination against rabies should be given to the first dog/cat at the age of 2-3 months.

🗖 Right 🗖 Not right

4.8 When the first vaccination is carried out, it is not necessary to re-vaccinate against rabies every year.

🗖 Right 🗖 Not right

4.9 If you inject the rabies vaccine to your pets by yourself, the vaccine should be stored in an ice box or refrigerator.

🗖 Right 🗖 Not right

4.10 Sick animals can also be vaccinated.

🗖 Right 🗖 Not right

4.11 Cropping of the dog's ears and tail will help prevent dogs from contracting the disease

🗖 Right 🗖 Not right

**Part 5 Suggestions for rabies prevention and control**

Suggestion(s) for government agency.............................................................

Suggestion(s) for other animal owners.....................................................

**Table S1** Questionnaire scoring table for knowledge, attitude and practice of dog owners regarding rabies control in Thailand

| ***Answers*/Knowledge** | 4.1 | 4.2 | 4.3 | 4.4 | 4.5 | 4.6 | 4.7 | 4.8 | 4.9 | 4.10 | 4.11 |  |
| --- | --- | --- | --- | --- | --- | --- | --- | --- | --- | --- | --- | --- |
| *Right* | 0 | 0 | 1 | 1 | 0 | 1 | 1 | 0 | 1 | 0 | 0 |  |
| *Not right* | 1 | 1 | 0 | 0 | 1 | 0 | 0 | 1 | 0 | 1 | 1 |  |
|  |  |  |  |  |  |  |  |  |  |  |  |  |
| ***Answers*/**  **Attitudes** | 3.1 | 3.2 | 3.3 | 3.4 | 3.5 | 3.6 | 3.7 | 3.8 | 3.9 | 3.10 | 3.11 |  |
| *Strongly agree* | 5 | 5 | 5 | 1 | 5 | 1 | 1 | 5 | 5 | 1 | 5 |  |
| *Agree* | 4 | 4 | 4 | 2 | 4 | 2 | 2 | 4 | 4 | 2 | 4 |  |
| *Unsure* | 3 | 3 | 3 | 3 | 3 | 3 | 3 | 3 | 3 | 3 | 3 |  |
| *Disagree* | 2 | 2 | 2 | 4 | 2 | 4 | 4 | 2 | 2 | 4 | 2 |  |
| *Strongly disagree* | 1 | 1 | 1 | 5 | 1 | 5 | 5 | 1 | 1 | 5 | 1 |  |
|  |  |  |  |  |  |  |  |  |  |  |  |  |
| ***Practices*** | 2.1 | Score | 2.2 | Score | 2.4 | Score | 2.5 | Score | 2.6 | Score | 2.8 | Score |
| *Answers* | *YesAnnually* | 2 | *Never* | 0 | *Never* | 0 | *No* | 0 | *No* | 0 | *No* | 0 |
|  | *YesNotAnnually* | 1 | *Yes* | 1 | *Ever* | 1 | *Yes* | 1 | *Yes* | 1 | *Yes* | 1 |
|  | *No* | 0 |  |  |  |  |  |  |  |  |  |  |

**Table S2** Descriptive statistics of the overall score of knowledge, attitude and practice of dog owners regarding rabies control in Thailand

| **Score** |  | **High risk areas** | **Low risk areas** | **Total** |
| --- | --- | --- | --- | --- |
|  | **Number of respondents** | **243** | **233** | **476** |
| Knowledge  (full score = 11) | Mean (SD*) | 8.12 (1.74) | 8.28 (1.54) | 8.20 (1.64) |
| Attitude  (full score = 55) | Mean (SD) | 42.36 (3.99) | 42.53 (4.06) | 42.44 (4.02) |
| Practice  (full score = 7) | Mean (SD) | 4.54 (1.51) | 4.36 (1.48) | 4.45 (1.50) |

*SD = Standard deviation
